# Medical Opinion and Movement

**Published:** 1907-04-27

**Authors:** 


### Medical Opinion and Movement.

*A propos* of hospital abuse and the recent endeavours in this country to restrict it, it is interesting to note that similar troubles are also encountered in the hospitals of Paris. The Board of "Assistance Publique" has just published a notice that consultations in the hospitals and dispensaries are reserved exclusively for patients without any means, the necessitous or indigent. Everyone applying to the hospital must prove by a card from the "Bureau de Bienfaisance," his identity, his residence in Paris, and his poverty. Apart from urgent cases, the doctors and the administrative officers should refuse medical attendance to anyone whose profession or address does not indicate poverty and a right to gratuitous attention. The Board will make domiciliary visits to verify the declarations of patients and will prosecute for the recovery of expenses and for the repression of abuses.

In a paper entitled "The Alleged Increase of Insanity," Mr. Noel A. Humphreys has endeavoured to solve the problem whether there is an actual increase of insanity in the community. The question is considerably hampered owing to the incompleteness of the returns of the Lunacy Commissioners on certain important points, such as the proportion of first admissions to asylums which have already been reported upon as living in workhouses or with relatives. Nevertheless, his conclusions are reassuring. He is able to show that two important factors are largely and probably entirely responsible for the continual increase in cases of certified insanity. These are, the increasing tendency to draw on the reserve of mental unsoundness hitherto outside the aggregate of actually certified cases, owing to an increased stringency in the standard of mental soundness; and the accumulation of the insane owing to the diminished death-rate and the prolonged stay of patients in the asylums.

A case of considerable medico-legal interest has recently been decided in France by the First Chamber of the Civil Tribunal. The plaintiff, a certain Madame Dury, entered the Hospital Lariboisière to undergo an operation upon the right kidney. The surgeon performed, however, a supravaginal hysterectomy. The patient sued the chief surgeon, Dr. Hartmann, and his assistant, Dr. Lecène, for 10,000 francs damages, on the ground that the operation was performed by the assistant instead of by the chief surgeon, Dr. Hartmann; that it was done without the consent of her husband; and that it was a different operation from what she had agreed to, and in her opinion quite unjustifiable. The Tribunal gave judgment for the defendants. It is probable that this judgment will be appealed against and the case taken to a higher court. In a case decided before the English courts, a nurse agreed to undergo laparotomy for removal of an ovarian cyst. In the course of the operation the surgeon performed total hysterectomy with removal of the appendages. The patient brought an action against the surgeon and obtained heavy damages.

The last meeting of the Clinical Society was productive of some very useful and interesting papers. Mr. Lawrie McGavin brought forward his method of filigree implantation for the cure of cases of very large or recurring inguinal herniæ. The author has already identified himself with the subject by his successful treatment of large abdominal herniæ by Bartlett's method of filigree implantation, and he now appears to have been equally successful in its application to inguinal hernia. At the same meeting Mr. G. H. Makins and Mr. Percy Sargent gave the results of the treatment of twenty-five cases of acute appendicular peritonitis by injections of a multivalent serum prepared from different strains of bacillus coli. The serum was prepared by Dr. H. H. Dale by inoculations of a horse with the strains separated by Dr. Dudgeon from different cases. Eight strains were taken from cases of puerperal fever and seven strains from cases of peritonitis, and other strains of unknown origin were also injected. In all cases the cultures injected were killed by the addition of 0.1 per cent. chinosol. The twenty-five cases treated were all of a severe type. Nine of these, or 36 per cent., recovered. It is further stated that marked improvement occurred in all, the toxic symptoms abated, the infected area became localised, and in the fatal cases the end was delayed beyond the usual period of the disease.

The house-fly has for some time past been regarded with serious suspicions by the sanitarian, and, in view of our knowledge of the spread of tropical diseases by the winged insects of the warmer climates, it has become increasingly probable that the house-fly is also responsible for the dissemination of infectious diseases. The bacteriologist to the Govan Town Council has been carrying out a series of experiments to ascertain the infective capacity of the fly. Flies were made to crawl over freshly prepared agar-agar contained in sterile Petri dishes. The dishes were then placed in an incubator at the normal temperature of the body, and at the end of twenty-four hours numerous colonies of micro-organisms appeared on the surface of the media. These micro-organisms were found on microscopical examination to be such as are associated with the presence of sewage and decomposing organic material. The question arises whether the summer diarrhœa of children may not be in a large measure due to contamination of the milk in this way by flies. This question is receiving the serious consideration of the Health Committee of Liverpool. At their instigation Professor Ronald Ross is investigating the life history of flies, especially with a view to ascertain their breeding place. One of the most important measures to restrain their activity is the removal of all decomposing refuse as rapidly as possible from the dwelling. Ash-pits should be replaced by proper sanitary bins, and people should be taught the necessity of burning all domestic refuse liable to decomposition and putrefaction.

